# Development and initial validation of the toxic personality scale: exploring reliability, structure, and convergent validity

**DOI:** 10.1186/s41155-026-00383-4

**Published:** 2026-02-20

**Authors:** Abdulselami Sarigül, Mehmet Emin Turan, Abdulmohsen Mohammed Abdullah Alkhulayfi, Juan Gómez-Salgado, Murat Yıldırım

**Affiliations:** 1 Department of Sociology, Faculty of Science and Letters, Ağrı İbrahim Çeçen University, Ağrı, Türkiye; 2 Department of Psychology, Faculty of Science and Letters, Ağrı İbrahim Çeçen University, Fırat Mahallesi Yeni Üniversite Caddesi No: 2 AE/1 04100, Merkez, Ağrı, Türkiye; 3 Department of Business Administration, Faculty of Economics and Administration, King Abdulaziz University, Jeddah, Saudi Arabia; 4 Department of Sociology, Social Work and Public Health, Faculty of Labour Sciences, University of Huelva, Huelva, 21071 Spain; 5 Safety and Health Postgraduate Program, Universidad Espíritu Santo, Guayaquil, 092301 Ecuador; 6 Psychology Research Center, Khazar University, Baku, Azerbaijan

**Keywords:** Toxic personality scale, Narcissism, Validity, Reliability, Turkish young adults

## Abstract

**Background:**

Toxic personality traits, which encompass behaviours and attitudes that negatively impact interpersonal relationships and personal well-being, have gained increasing attention in psychological research. However, a significant gap remains in developing reliable and culturally relevant measures to assess these traits.

**Objective:**

This study addressed a research gap by developing a new measure for determining toxic personality traits, the Toxic Personality Scale (TPS), and evaluating its reliability and validity across two samples of young Turkish adults.

**Methods:**

To develop the scale, two studies were conducted. Study 1 included 389 undergraduate students (78.66% male, 21.34% female) aged 18 to 48 years (M = 21.45, SD = 4.16). Study 2 involved 158 participants (72.78% female, 27.22% male) aged 18 to 35 years (M = 21.28, SD = 2.62). Participants completed the TPS and the Short Dark Triad through an online survey.

**Results:**

The exploratory and confirmatory factor analyses suggested that the TPS scale was unidimensional, with six items demonstrating a good internal consistency reliability estimate. The TPS also showed a significant positive relationship with the measure of narcissism.

**Conclusion:**

The findings suggest that the TPS is a valid and reliable tool for assessing toxic personality traits. These results support the TPS as a valuable instrument for use in both research and clinical settings to better understand and identify toxic personality traits in individuals.

## Introduction

Personality types exhibit different positive and negative characteristics as a result of genetics, age, physical structure, shock experiences, and social relationships, and these changes are explained by the general system theory (Plomin & Nesselroade, 1990; Üngüren, 2016). Although some of these changing personality traits (feeling valuable and self-esteem) are positive and beneficial for the individual and society, some personality traits (such as hedonistic and self-centred, scheming, manipulative, sarcastic, dishonest, hurtful) (Campbell, 2021; Twenge & Campbell, 2015) can harm people’s emotions and self-in bilateral relations and prevent well-being (Sarıgül, 2023). These personality traits, which are centred on pleasure and self and spread throughout society, are considered an epidemic (Twenge & Campbell, 2015). This epidemic can focus on pleasure and self, as well as manipulating others to make them feel bad for their desires (Sarıgül, 2023; Set, 2020; Twenge, 2013). These negative human characteristics, which are formed to manipulate or make others feel bad in a relationship, have transformed the forms of relationships between people in the social structure. To identify these transformation dimensions, various scales have been developed to understand relationship styles such as interpersonal exploitativeness and managing the emotions of others (Austin et al., 2018; Brunell et al., 2013). These types of scales aim to identify individual-based relationship problems that occur in society. Because relationships with people who have negative personality traits can have negative psychological and social consequences (Siria et al., 2021; Sosic-Vasic et al., 2024; Zhu et al., 2024).

A toxic relationship, which is one of the negative relationship styles, can negatively affect people’s social lives by affecting them mentally and physically (Doğan & Sapmaz, 2012). People with toxic personality traits can cause harm to others through interaction (Firestone & Catlett, 2018). That is why there has been a growing interest in understanding toxic people lately (Akbulut & Yavuz, 2022; Arda & Kanten, 2023; Campbell, 2021; Karaca & Aksoy, 2024; Suster, 2023; Yalçınsoy & Işık, 2018). The increase in negative situations caused by individuals who exhibit toxic characteristics in social life, and the fact that they are frequently mentioned by experts on social media platforms, has transformed the problem from an individual issue to a psychosocial issue (Sarıgül, 2023).

Toxic, in chemistry and medical science, can be used in the sense of poison that can affect a living being and cause disability and death due to the toxic effect of any substance (Gangel, 2008). Different behaviours such as toxic leadership, unethical behaviours in bilateral relations, manipulation, intimidation, negative behaviours, micromanagement, and bullying can also occur in personality (Pelletier, 2010; Spagnoli et al., 2020). Certain characteristics of a toxic person are listed as; using manipulation techniques, making people feel incomplete and flawed, criticising and judging other people, making people feel bad, causing discomfort, constantly finding faults, not hesitating to lie for the sake of their interests, not liking to apologise and not preferring apologies in communication, always prioritizing their interests and ignoring the needs of other people (Sarıgül, 2023). There is evidence that many of the mental health problems people experience stem from negative relationships with others (Forth et al., 2021; Öztürk et al., 2016). On the one hand, studies in the field focus on positive psychology, and on the other hand, on the measurement and conceptualization of negative human characteristics and the relationships that arise from them (Afacan Fındıklı et al., 2019; Jarukasemthawee et al., 2024; Jones & Paulhus, 2014). In this study, the conceptualization, development, validity and reliability study of the Toxic Personality Scale (TPS), which affects people’s communication with others and makes them unproductive, was conducted. According to the previous studies, there is no scale in the literature on toxic personality, and most of the measurements obtained are aimed at assessing toxic leadership Schmidt 2008a, b; Labrague et al. 2020). In addition, scales related to toxic relationships do not provide an absolute assessment to fully conceptualize and define a toxic personality. For this reason, we introduced the concept of toxic personality in the study and took the discussion beyond toxic leadership.

There is an important reason for introducing the TPS to the literature beyond the concept of toxic leadership. Namely, while the literature on toxic people focuses only on typical leaders, our study focuses on individuals who show toxic characteristics in social life. By providing evidence based on the individual negative aspects of toxic people, the study can provide a broad framework based on the full disclosure of the individual characteristics of toxic people. Therefore, the main objectives of the study are (1) to discuss empirical and conceptual aspects of the measured toxic personality, (2) to propose a new definition of toxic personality, to report the data obtained about the TPS, and to make suggestions for future studies of toxic personality.

The emergence of the toxic person is attributed to the weakening of social bonds and the decrease in control in the social structure, and it is stated that this situation produces the toxic type and causes discontent (Sarıgül, 2023). Previous studies provide evidence that individuals cannot fulfil their duties and responsibilities in toxic environments (Demir, 2020). However, it is reported that toxic parents cause unrest by damaging the environment of trust, which is the basic duty of the family (Campbell, 2021). Toxic people can be defined as people who humiliate, ridicule, behave unpleasantly, use, make others feel defective, harm, lie, judge, act superior, act cunningly, and do these things constantly and consistently with bad intentions (Campbell, 2021; Firestone & Catlett, 2018; Sarıgül, 2023).

Toxic personality refers to a persistent pattern of maladaptive traits and interpersonal behaviors that undermine psychological well-being and relational functioning. These traits often manifest as emotional volatility, manipulation, hostility, and boundary violations, and are typically resistant to feedback or change.

From a trait-based perspective, a toxic personality can be conceptualized as an extreme configuration of high neuroticism, low agreeableness, and low conscientiousness within the Five-Factor Model (McCrae & Costa, 1999). These traits correspond closely with maladaptive domains identified in the ICD-11 and DSM-5 alternative model for personality disorders—particularly antagonism/dissociality and negative affectivity, with contributions from disinhibition, detachment, and anankastia depending on context.

While toxic traits may not meet diagnostic criteria for personality disorders, they produce significant interpersonal harm and are often reinforced by short-term relational gains. Recognizing toxic personality as a dimensional construct allows for early identification and targeted intervention, especially in clinical, educational, and organizational settings.

We have found that a toxic personality is a distinct human trait in a pattern that is separate from the other negative personality traits specified in DSM-5. The toxic personality exhibits patterns that are similar to but different from the narcissism traits specified in DSM-5. These patterns emerge through interactions with others in society (Sarıgül, 2023) and are characterized by attitudes and behaviors intended to undermine or harm the other person emotionally. Unlike narcissistic individuals, who primarily seek to elevate themselves, toxic individuals may actively cause others to feel worthless or self-loathing. They often aim to erode the other person’s sense of self by demeaning or invalidating their identity. In this sense, the toxic personality reflects more complex and destructive dynamics than narcissistic traits. Furthermore, engaging with toxic individuals can result in emotional abuse and have detrimental effects on one’s psychological well-being. Especially in emotional abuse caused by toxic family members, individuals’ identity, personality, and psychological development processes can be damaged (Campbell, 2021). For this reason, defining and measuring the toxic personality type may provide opportunities for effective intervention in individuals’ psychopathologies. In previous studies, there is a scale related to toxic leadership (Schmidt 2008a, b). However, no scale has been found that attempts to identify the characteristics of toxic individuals that cause people to feel bad about themselves and hate their existence as a result of their relationships.

### Current study

The characteristics of a narcissist and a toxic individual are often confused with each other due to their similarities. It is possible to make a conceptual distinction between the two as follows. The difference between a toxic personality and a narcissistic personality is that narcissists do everything to feel good and are at the centre of themselves (Giacomin & Jordan, 2016). However, a toxic personality does this to make the other person feel bad. While a narcissist establishes a relationship focused on himself, a toxic individual establishes a relationship aimed at making the other person feel bad. Indirectly, a toxic personality can be defined as a personality type that establishes relationships focused on power relations in society and in a manipulative manner, making the other person feel bad. Recognising a toxic personality can provide opportunities for effective intervention in individuals’ psychopathologies.

Developing a measure of toxic personality is important as it fills a significant gap in personality assessment. While many existing measures focus on broad traits, such as the Big Five (Goldberg, 1992), or specific maladaptive traits like narcissism, Machiavellianism, and psychopathy—often collectively referred to as the Dark Triad (Jones & Paulhus, 2014)—these scales do not fully encapsulate the behaviors and characteristics associated with toxic personality traits. It is also important to differentiate between a toxic personality and a dark triad personality. The dark triad consists of three overlapping but separate traits: narcissism, Machiavellianism, and psychopathy, all of which are characterized by a lack of empathy and a strong tendency to manipulate others. While individuals with a toxic personality may exhibit some manipulative behaviors, they do not necessarily display the pervasive and strategic manipulation seen in the dark triad. Furthermore, toxic individuals may retain some level of empathy, whereas dark triad personalities often exhibit very low or even no empathy at all. This distinction suggests that while both toxic and dark triad personalities can be socially harmful, the toxic personality is more situational and relational, whereas the dark triad traits are more deeply ingrained and pervasive (Jones & Paulhus, 2014; Sarıgül, 2023; Suster, 2023).

Another key distinction we also make in this study is between toxic personality and antisocial personality. While both involve socially detrimental behaviors, they manifest in distinct ways. A toxic personality is primarily defined by harmful interpersonal behaviors, particularly in relationships and workplace settings, such as manipulation, persistent negativity, and excessive criticism. In contrast, antisocial personality is characterized by a pervasive and chronic pattern of manipulation, exploitation, and violation of others’ rights, often without remorse. This condition is more closely linked to criminality and overt aggression (Black, 2017; Sarıgül, 2023; Suster, 2023).

By developing the TPS, we aim to provide a tool whose scores can be interpreted reliably and validly for the purpose of identifying and measuring toxic personality traits. Understanding these traits is important, as they can significantly impact social and professional interactions, often leading to interpersonal conflict, reduced workplace cohesion, and diminished psychological well-being. Despite growing interest in toxic personality, there remains a lack of standardized instruments to assess these traits systematically. The TPS seeks to fill this gap by offering a targeted measure that captures personality characteristics known to disrupt social harmony, strain relationships, and contribute to negative emotional and psychological outcomes.

## Methods for study 1

The aim of Study 1 was to develop the TPS by exploring the psychometric properties of the items, the underlying factor structure, and its associations with narcissism.

### Participants

Three hundred eighty-nine undergraduate students (78.66% male, 21.34% female) participated in Study 1. Ages ranged from 18 to 48 years, with a mean age of 21.45 years (SD = 4.16). In terms of academic classification, more than half of the participants (50.90%) were freshmen, followed by sophomores (24.68%), juniors (9%), seniors (14.14%), and post-graduate students (1.29%). The majority of participants (81.50%) indicated a medium level of academic achievement, while 12.30% reported high achievement, and 6.20% reported low achievement. Concerning socioeconomic status, most participants (79.20%) perceived it as average, with 18.80% considering it below average and 2.10% regarding it as above average.

### Development of the item pool for the toxic personality scale

The development of the TPS followed a multi-step process to ensure its conceptual clarity, empirical validity, and psychometric soundness. Given the limited theoretical and empirical groundwork on toxic personality, we adopted a systematic approach involving item generation, expert review, refinement, and preliminary validation.

Regarding item generation, we constructed an initial item pool by conducting an extensive literature review across multiple databases, including Web of Science, PubMed, PsycINFO, and Google Scholar. Our search strategy focused on the term “toxic personality” as well as related constructs such as manipulation, deceitfulness, emotional exploitation, and interpersonal toxicity. We also examined references from relevant articles to capture various theoretical and empirical perspectives on toxic personality.

Given the lack of a unified definition, we sought to integrate diverse perspectives on toxic traits from psychological, counselling, and sociological literature. The research team, consisting of experts in psychology, counselling, and sociology, engaged in multiple brainstorming sessions to identify key characteristics commonly associated with a toxic personality, such as dishonesty, manipulation, lack of empathy, and interpersonal exploitation. Based on these discussions and literature synthesis, an initial pool of 50 items was generated, each designed to capture toxic personality.

Concerning expert review and refinement, to ensure content validity, the initial pool of 50 items underwent a review by an independent panel of three subject-matter experts, including clinical psychologists, social psychologists, and psychometricians. Experts were asked to evaluate each item for relevance, clarity, and redundancy. Based on expert feedback, 20 items were removed due to redundancy, lack of clarity, or insufficient representation of the construct. Several items were also reworded for clarity and precision. This refinement process resulted in a final set of 30 items, each succinctly and accurately capturing toxic personality, which was subsequently tested in Study 1.

### Measures

#### Toxic Personality Scale (TPS)

The final version of the scale consisted of 30 items, each rated on a 5-point Likert-type scale ranging from 1 (strongly disagree) to 5 (strongly agree), where higher scores indicated greater levels of toxic personality. A sample item is “I frequently tell lies to manipulate situations.” A full list of items in English (Appendix A) and Turkish (Appendix B) is provided in the Appendix.

### Other measure

#### The Short Dark Triad (*SD3-T*) 

The Short Dark Triad (SD3-T; Jones & Paulhus, 2014) is a short proxy measure used to assess personality traits. The SD3-T comprises 27 self-reported items distributed across three subscales: narcissism, Machiavellianism, and psychopathy, each consisting of 9 items. For this study, we specifically focused on the narcissism subscale. Participants were required to answer all items on a 5-point Likert-type scale varying from 1 (strongly disagree) to 5 (strongly agree). The English version of the SD3-T was translated into Turkish by Özsoy et al. (2017).

### Procedure

In this study, participants were recruited through convenience sampling using an online survey platform. The survey link was distributed via social media and university mailing lists to reach a broad sample of individuals who met the inclusion criteria: being over the age of 18, residing in Turkey, and providing informed consent to participate. No compensation was offered for participation. Ethical approval for this study was obtained from the Ethics Committee of Ağrı İbrahim Çeçen University (Approval Number: E-95531838-050.99-89779). All participants provided informed consent prior to their participation in the study, after being adequately informed about the purpose, procedures, potential risks, and their right to withdraw at any time without any penalties. Participant confidentiality and anonymity were assured by assigning codes instead of using names or other identifying information, and all data were kept secure and confidential in password-protected files in compliance with ethical guidelines.

### Data analysis

Internal consistency reliability was estimated to assess the reliability of the TPS using Cronbach’s alpha and split-half reliability. Exploratory factor analysis (EFA) was performed on Study 1 data to uncover the underlying factor structure of the scale. Skewness and kurtosis values were examined to assess the normality assumption, with values within ± 2 and ± 7, respectively, considered acceptable for most statistical analyses (Curran et al., 1996). Pearson correlation was carried out to explore the correlation between toxic personality and narcissism. All analyses were conducted using SPSS and AMOS for Windows.

## Results for study 1

### Item analysis

Mean, standard deviation, item-total correlations, Cronbach’s alpha if the item is deleted, and factor loadings for the scale’s items are presented in Table 1. Items’ total correlation ranged between 0.54 and 0.56 for Sample 1 and 0.51—0.62 for Sample 2. In both samples, the factor loadings varied from 0.57 to 0.72, showing good to excellent factor loadings (Comrey & Lee, 2013). The descriptive analysis indicated that both skewness and kurtosis values fell within acceptable thresholds, suggesting approximate normality of the variables. Specifically, skewness ranged from 0.68 to 1.72 in Study 1 and from 0.88 to 1.64 in Study 2, while kurtosis ranged from − 0.26 to 2.67 in Study 1 and from 0.09 to 2.89 in Study 2.

**Table 1:** Mean, standard deviation, reliability and factor loadings for the scale’s items

Item	Study 1 (*n* = 389)							Study 2 (*n* = 158)						
	M	SD	IC	CD	EFA loading	Skewness	Kurtosis	M	SD	IC	CD	CFA loading	Skewness	Kurtosis
Item 1	1.65	0.85	0.54	0.76	0.70	1.72	2.67	1.71	0.93	0.60	0.79	0.66	1.64	2.89
Item 2	1.75	0.94	0.56	0.76	0.72	1.47	2.21	1.69	0.92	0.51	0.80	0.57	1.56	2.47
Item 3	2.33	1.08	0.56	0.76	0.71	0.68	-0.26	2.16	1.09	0.61	0.78	0.70	0.88	0.09
Item 4	2.07	1.07	0.54	0.76	0.69	1.15	0.89	2.06	1.19	0.58	0.79	0.66	1.06	0.17
Item 5	2.13	1.07	0.54	0.76	0.69	0.97	0.38	2.01	1.01	0.62	0.78	0.71	0.98	0.42
Item 6	1.79	1.01	0.55	0.76	0.71	1.48	1.92	1.85	1.02	0.58	0.79	0.64	1.21	0.95

*M *mean, *SD *standard deviation, *IC *item-total correlations, *CD *Cronbach’s alpha if item deleted

### Exploratory factor analysis

A maximum likelihood factor (ML) analysis with Promax rotation was conducted on the initial set of 30 items. ML was selected due to its suitability for approximately normally distributed data and its capacity to provide statistical tests of model fit (Fabrigar et al., 1999). Promax, an oblique rotation method, was chosen to accommodate the theoretical expectation that the underlying psychological constructs would be interrelated. Eight components with eigenvalues above 1 were identified, explaining approximately 61% of the shared variance. However, certain items did not contribute to a clear and empirically justifiable structure, displaying low communalities and/or cross-loadings such as rough factor loadings in more than one factor. Communalities below 0.4 were considered low (Nunnally, 1978). Therefore, we sought to have factor loadings above 0.5 (Byrne, 2013). Cross-loadings were identified when the difference between loadings on different components exceeded 0.20, accounting for approximately 4% of the variance. Through an iterative process involving the removal of one item at a time and re-evaluation of the factorial structure each time, 24 items were eliminated. This final solution yielded a one-factor structure, resulting in a final version of the scale with six items, with an eigenvalue of 2.97, accounting for 49.42% of the variance. The factor loadings ranged between 0.63 and 0.65, which are presented in Table 1.

### Convergent validity

Using Study 1 data, we calculated correlation coefficients between toxic personality and narcissism using Study 1 data. The correlation results indicated a significant positive relationship between scores of toxic personality and narcissism (*r* = 0.37, *p* < 0.01). The effect size for this correlation was medium, following Cohen’s (1988) criteria, where *r*≥0.5 signifies a large effect size, 0.3 ≤ *r*<0.5 represents a moderate effect size, and 0.1 ≤ *r*<0.3 shows a small effect size.

## Methods for study 2

The aim of Study 2 was to verify the factor structure of the TPS measure.

### Participants

The sample comprised 158 respondents (72.78% female, 27.22 male) aged from 18 to 35 years (*M* = 21.28 years, *SD* = 2.62). Nearly two-thirds of the participants (60.76%) were sophomores, followed by freshmen (28.48%), seniors (3.16%), juniors (1.27%), post-graduate students (3.16%) and preparatory class (3.16%). Participants predominantly indicated a medium level of academic achievement (81.65%), while 15.82% reported high achievement and 2.53% reported low achievement. Regarding socioeconomic status, the majority of participants (77.22%) perceived it as average, with 20.25% considering it below average and 2.53% regarding it as above average.

### Measures

The participants completed only the TPS measure described in Study 1.

### Procedure

The procedure was identical to that outlined in Study 1.

### Data analysis

In line with expert guidelines, we used a minimum threshold of 150 participants for conducting Confirmatory Factor Analysis (CFA) under conditions of normal distribution and complete data (Muthén & Muthén, 2002). CFA, executed with SPSS-AMOS on Study 2 data, aimed to validate the TPS factor structure. To evaluate the goodness of fit, we presented the relative chi-square (CMIN/DF) in conjunction with the chi-square and degrees of freedom. Additionally, we considered the Comparative Fit Index (CFI), Incremental Fit Index (IFI), Non-Normed Fit Index (NNFI), Standardized Root Mean Square Residual (SRMR), and Root Mean Square Error of Approximation (RMSEA) as recommended by Hu and Bentler (1999). Acceptable fit is denoted by a CMIN/DF less than 2 or 3, IFI, CFI, and NNFI of at least 0.90, RMSEA index between 0.05 and 0.08, and SRMR less than 0.08.

## Results for study 2

### Confirmatory factor analysis

**Fig. 1:**
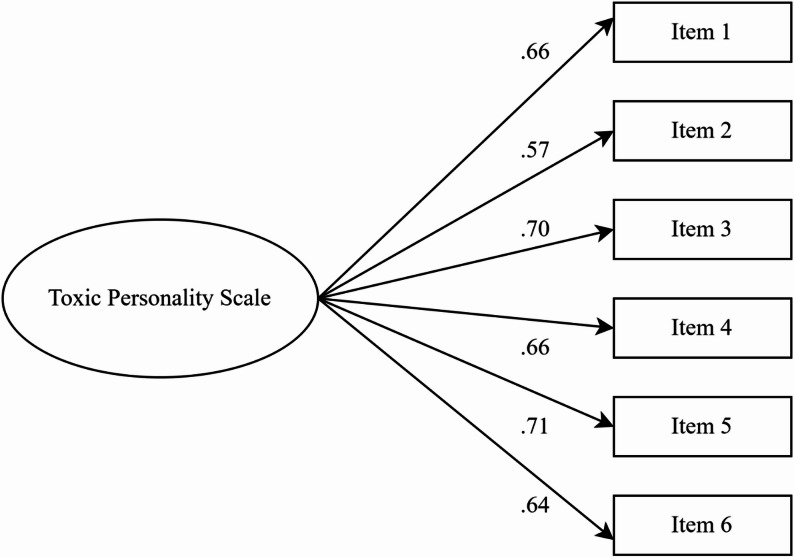
Standardized factor loadings of the TPS

The EFA results were validated using the second dataset, and a CFA was performed to assess the hypothesized one-factor structure of TPS items. In the Study 2 sample, the CFA demonstrated satisfactory goodness-of-fit indices [χ2(9) = 21.66, *p* = 0.10, CMIN/DF = 2.41, IFI=0.95, NFI=0.92, CFI=0.95, SRMR=0.049, and RMSEA=0.10]. While the RMSEA value for the model is marginally above the commonly accepted threshold of 0.08, this may be influenced by the model’s small degrees of freedom and sample size (Kenny et al., 2015), which can sometimes produce artificially high RMSEA estimates and incorrectly suggest poor model fit. Nevertheless, other fit indices fell within acceptable ranges, and the results collectively indicate that the one-factor model adequately fits the observed data. The standardized factor loadings were adequate, ranging from 0.57 to 0.71, as presented in Table 1; Fig. 1.

### Internal consistency reliability

The internal consistency reliability of the six items was assessed for both samples using Cronbach’s alpha and split-half reliability. A good Cronbach’s alpha coefficient for six items was observed, with values of α = 0.80 in Sample 1 and α = 0.82 in Sample 2. Additionally, split-half reliability was also reported as an additional indicator of internal consistency. It showed a correlation of 0.69 in Sample 1 and 0.70 in Sample 2. In addition to Cronbach’s alpha, we also calculated the composite reliability (CR) and average variance extracted (AVE) for each latent construct. CR values above 0.70 and AVE values above 0.50 are considered indicative of acceptable internal consistency and convergent validity (Fornell & Larcker, 1981). For the toxic personality construct, the CR values were 0.80 in Sample 1 and 0.82 in Sample 2, indicating good reliability. However, the AVE values were 0.39 in Sample 1 and 0.43 in Sample 2, falling below the recommended threshold and suggesting limited convergent validity.

## Discussion

To the best of our knowledge, the TPS is the first measurement tool specifically designed to assess toxic personality traits. The present study aimed to develop the TPS and evaluate its factorial structure, reliability, and validity using two samples of young Turkish adults. This new measure is intended to enhance our understanding of toxic personality traits. The findings support the construct validity of the TPS. Exploratory factor analysis revealed that all items had high loadings on a single factor, indicating that the TPS is a unidimensional measure. Confirmatory factor analysis further confirmed this single-factor structure. Reliability analysis showed that the TPS has good internal consistency (Sample 1: α = 0.80; Sample 2: α = 0.82), indicating satisfactory reliability. Additionally, the TPS demonstrated convergent validity through positive correlations with measures of narcissism. Overall, the results suggest that the TPS is a reliable and valid tool for assessing toxic personality traits in young adults.

A crucial aspect is the distinction between a toxic personality and other negative personality traits, such as the dark triad. Whilst narcissistic, machiavellian, and psychopathic disorders primarily centre on self-evaluation and manipulation for personal gain (Jones & Paulhus, 2014), toxic personality involves a distinct relational component where the destruction of the other person’s self-esteem is a principal objective (Sariğül, 2023). Furthermore, the cultural implications of a toxic personality must be considered. For instance, certain studies suggest that toxicity in interpersonal relationships may vary depending on cultural context and social norms (Jarukasemthawee et al., 2024). Therefore, these findings should be interpreted within the cultural context in which the scale was developed. In Turkish society, collectivist values, relational sensitivity, and hierarchical social structures may influence how individuals perceive and report toxic traits. Behaviors such as assertiveness or emotional expressiveness may be viewed as disruptive or disrespectful depending on situational norms, while indirect forms of control or manipulation may be more socially tolerated. Such cultural dynamics can influence both the expression and self-reporting of toxic traits, suggesting the importance of culturally grounded interpretation when evaluating personality constructs.

Measures like the TPS generally evaluate toxic leadership (Schmidt 2008a, b) and other personality traits (Jones & Paulhus, 2014; Sleep et al., 2020). In contrast to similar measure studies, different personality characteristics and toxic relations related to toxic personality are highlighted and evaluated in this study. This has made it possible to evaluate the toxic personality on a broader understanding through the different personality characteristics and toxic relationships. Conversely, the present study can contribute to personality research by providing a valid and reliable measure that will enable researchers and practitioners to evaluate the toxic personality.

### Implications

The findings of this study hold important implications for personality research. The TPS offers several advantages for both researchers and practitioners. Firstly, despite its brevity, the scale has demonstrated adequate psychometric properties, making it a practical tool for large-scale data collection. Secondly, its ease of use allows researchers to efficiently gather data in a short amount of time (Gosling et al., 2003). Thirdly, the TPS provides a structured means of evaluating toxic personality, enabling a more precise understanding of its characteristics and effects, as well as complementing existing personality frameworks such as the Dark Triad (Paulhus & Williams, 2002) and the Big Five Personality Traits (McCrae & Costa, 1997). Additionally, the TPS can contribute to cross-cultural studies by helping researchers examine differences in toxic personality traits across diverse populations. Moreover, in organizational settings, the TPS can help identify the negative impact of toxic personality traits on workplace well-being. In therapeutic contexts, it can serve as a guiding tool for personality-based interventions, facilitating the development of targeted strategies to measure toxic personality traits. Clinicians and counselors can use the TPS to better understand and assess individuals exhibiting harmful interpersonal behaviors, which can inform tailored intervention strategies aimed at reducing relational conflict and promoting healthier social dynamics.

Given that research on toxic personality remains relatively studied, the TPS has the potential to advance the field significantly. Future studies utilizing the TPS in conjunction with other established personality measures, such as the Dark Triad or the Big Five Personality Traits, could provide a better understanding of the underlying mechanisms of toxic personality. Moreover, the TPS may serve as a useful tool for examining the influence of toxic personality on various life outcomes and its role in personal and social functioning. Understanding how toxic personalities are linked to and explain life outcomes can assist researchers and practitioners in developing effective interventions to manage negative personality traits and enhance wellness. For example, given the link between toxic personality and theoretically similar variables, practitioners can use the findings of the current research to determine the focal point of the intervention so that they can improve the life outcomes and wellness of individuals by managing their toxic personality characteristics.

### Limitations

The present study has several limitations that should be taken into account when examining the findings of the research. First, our preliminary findings suggest that the TPS demonstrates acceptable reliability and validity in assessing toxic traits among young Turkish adults. However, further research is needed to verify these initial results. While internal consistency was examined, one limitation remains. Test–retest reliability was not assessed during the original data collection, restricting conclusions about the temporal stability of TPS scores. Future studies should incorporate longitudinal designs to strengthen the scale’s psychometric properties. Also, in the current study, we examined the construct and convergent validity of the TPS. However, other forms of validity, such as discriminant and predictive validity, were not assessed. Future research should adopt broader validation strategies to better establish the scale’s theoretical distinctiveness and its capacity to predict relevant behavioral and psychological outcomes. Second, although the sample size of 150 participants can be considered adequate for the analyses conducted, the statistical power remains relatively modest, which may limit the strength and generalizability of the conclusions. Also, the reliance on a convenience sample consisting predominantly of young Turkish university students, coupled with a gender imbalance across the subsamples, further constrains the extent to which the findings can be generalized to broader populations. Although the sample size was adequate for initial scale development, future research should replicate the study with more diverse populations across different geographical, cultural, and socioeconomic backgrounds to strengthen the generalizability of the TPS. Third, the reliance on Likert-type data analyzed with maximum likelihood estimation may not fully capture the ordinal or nonlinear nature of the responses. Future research could employ alternative estimators (e.g., Weighted Least Squares Mean and Variance Adjusted, Robust Maximum Likelihood) or Rasch modeling to achieve more precise item calibration. Although Rasch analysis provides valuable evidence regarding item functioning and measurement precision, it was not applied in the present study due to the scope of the work, its grounding in classical test theory, and methodological familiarity. Future studies may benefit from incorporating Rasch modeling to further strengthen the psychometric evaluation of the scale. Fourth, the RMSEA value of 0.10, while partly attributable to the small number of items and limited degrees of freedom, may also reflect potential model misspecification. Readers should interpret this fit index cautiously, and replication with larger item pools and alternative estimators is recommended. Additionally, as severe imbalance can affect the stability and interpretability of the results, we did not perform measurement invariance tests across subgroups, which would be desirable to account for potential differences stemming from these sample disparities. Future research should address this limitation. Another limitation concerns the potential for response bias, particularly in the form of social desirability or impression management. Given the socially undesirable nature of toxic traits, participants may have underreported certain behaviors or attitudes. Future studies should incorporate validated measures of response bias to better account for this possibility and improve the accuracy of self-reported data. Finally, we found that there is a relationship between toxic personality and narcissism by using the cross-sectional method. Thus, this correlation between toxic personality and narcissism needs to be approached with great attention.

## Conclusion

This study developed and validated the measure of toxic personality as a brief, reliable, and unidimensional measure of toxic personality. The TPS provides a practical tool for researchers and practitioners to assess toxic traits, explore their development, and inform intervention strategies. Its brevity and good psychometric properties make it suitable for use in personality research, interpersonal, organizational, and clinical settings.

## Data Availability

The datasets generated during and/or analysed during the current study are available from the corresponding author upon reasonable request.

## Appendix 1: a full list of items in English

### Toxic personality scale

Please read each statement below carefully and select the option that best describes you and your personality.

1 = Strongly disagree

2 = Disagree

3 = Neutral

4 = Agree

5 = Strongly agree

**Table:**

1. I frequently tell lies to manipulate situations.	1	2	3	4	5
2. I often insult others to put them down.	1	2	3	4	5
3. When I am angry with someone, I deliberately hinder their progress.	1	2	3	4	5
4. I avoid apologising, even when I know I am wrong.	1	2	3	4	5
5. I enjoy manipulating others into doing things for me.	1	2	3	4	5
6. I believe I am skilled at deceiving others to get what I want.	1	2	3	4	5

The scale does not include any reverse-coded items. All items are summed to compute a total score, which ranges from 6 to 30. A higher score indicates a greater level of toxic personality traits.

## Appendix 2: a full list of items in Turkish

### Toksik kişilik ölçeği

Lütfen aşağıdaki her bir ifadeyi dikkatlice okuyun ve sizi ve kişiliğinizi en iyi tanımlayan seçeneği işaretleyin.

1 = Kesinlikle katılmıyorum

2 = Katılmıyorum

3 = Ne katılıyorum ne katılmıyorum

4 = Katılıyorum

5 = Kesinlikle katılıyorum

**Table:**

1. Durumları manipüle etmek için sık sık yalan söylerim.	1	2	3	4	5
2. Başkalarını küçük düşürmek için sık sık hakaret ederim.	1	2	3	4	5
3. Birine kızdığımda, kasıtlı olarak onun ilerlemesini engellerim.	1	2	3	4	5
4. Hatalı olduğumu bilsem bile özür dilemekten kaçınırım.	1	2	3	4	5
5. Başkalarını benim için bir şeyler yapmaları için manipüle etmekten hoşlanırım.	1	2	3	4	5
6. İstediğimi elde etmek için başkalarını kandırma konusunda yetenekli olduğuma inanıyorum.	1	2	3	4	5

Ölçekte ters kodlanan madde bulunmamakta olup, maddelerin tamamı toplanarak toplam puan elde edilmektedir. Toplam puan 6 ile 30 arasında değişebilir ve daha yüksek puan daha yüksek düzeyde toksik kişilik özelliğine işaret etmektedir

## Abbreviations

M: Mean
SD: Standard deviation
IC: Item-total correlations
CD: Cronbach’s alpha if item deleted
EFA: Exploratory factor analysis
CFA: Confirmatory factor analysis
TPS: Toxic Personality Scale
SD3-T: Short Dark Triad
CMIN/DF: Relative chi-square
CFI: Comparative fit index
IFI: Incremental fit index
NNFI: Non-normed fit index
SRMR: Standardized root mean square residual
RMSEA: Root mean square error of approximation
